# Alteration of functional connectivity despite preserved cerebral oxygenation during acute hypoxia

**DOI:** 10.1038/s41598-023-40321-3

**Published:** 2023-08-15

**Authors:** Marleen E. Bakker, Ismaël Djerourou, Samuel Belanger, Frédéric Lesage, Matthieu P. Vanni

**Affiliations:** 1 École d’Optométrie, Université de Montréal, 2500 Chem. De Polytechnique, Montréal, QC H3T 1J4 Canada; 2 Institute of Biomedical Engineering, École Polytechnique de Montréal, Montréal, Canada; 3 Labeo Technologies Inc, Montréal, Canada; 4 Montréal Heart Institute, Montréal, Canada

**Keywords:** Biological techniques, Neuroscience

## Abstract

Resting state networks (RSN), which show the connectivity in the brain in the absence of any stimuli, are increasingly important to assess brain function. Here, we investigate the changes in RSN as well as the hemodynamic changes during acute, global hypoxia. Mice were imaged at different levels of oxygen (21, 12, 10 and 8%) over the course of 10 weeks, with hypoxia and normoxia acquisitions interspersed. Simultaneous GCaMP and intrinsic optical imaging allowed tracking of both neuronal and hemodynamic changes. During hypoxic conditions, we found a global increase of both HbO and HbR in the brain. The saturation levels of blood dropped after the onset of hypoxia, but surprisingly climbed back to levels similar to baseline within the 10-min hypoxia period. Neuronal activity also showed a peak at the onset of hypoxia, but dropped back to baseline as well. Despite regaining baseline sO2 levels, changes in neuronal RSN were observed. In particular, the connectivity as measured with GCaMP between anterior and posterior parts of the brain decreased. In contrast, when looking at these same connections with HbO measurements, an increase in connectivity in anterior–posterior brain areas was observed suggesting a potential neurovascular decoupling.

## Introduction

A lack of oxygen in tissue (hypoxia) can occur in different ways, such as lack of atmospheric oxygen, inability of blood cells to bind oxygen, or a stroke. Research into the effects of hypoxia has received increased attention^1^ since 2020, after discovery that COVID-19 can cause severe respiratory syndrome^2^, and thus might have exposed millions of people to hypoxic episodes.

The brain response to hypoxia differs depending on the oxygen level, whether it is acute or chronic, continuous or intermittent, and as a function of age^3^. Hypoxia in the brain has a negative impact on perception, cognition and motor performance^4–6^. In cases of acute hypoxia, these changes have been shown to precede histopathological changes^3^.

At the physiological level, hypoxia influences brain hemodynamics in several ways. Studies have shown an increase of cerebral blood flow (CBF)^7–14^ as well as respiration rate^12,15^, depending on the severity of hypoxia. However, these adaptations were insufficient to counteract the decline in systemic oxygen saturation levels^6,8,9,16–18^ and cognition^4,5,19,20^ during hypoxic periods.

At the cellular level, changes in neuronal activity with both depolarization^21^ and hyperpolarization^22^ have been previously reported. However, the neuronal changes are spatially heterogeneous, showing differences not only per area, but also per neuron within area^3^, with areas that are important for respiratory functions being the most spared of the detrimental effects^23^. In general, the neuronal activity tends to decrease in hypoxic conditions. During severe hypoxia (6% oxygen), the EEG signal recorded in mice was largely suppressed, together with the respiration rate^15^, which the authors suggest indicates an impairment in consciousness. Furthermore, measurements using electrically evoked action potentials in vitro show that hypoxia abolishes the evoked action potentials (in extreme hypoxic conditions: 0% oxygen, 95% nitrogen and 5% CO2)^21^. Although it would stand to reason that hypoxia has a solely detrimental effect on neuronal activity given neuron’s dependence on oxygen for survival and firing ability, the effect of hypoxia on neuronal signals is far from universal and can have positive effects as well. For example, intermittent hypoxia can be an effective treatment for certain neurological dysfunctions, such as spinal cord injury^24^.

Overall, the short-term molecular mechanisms associated with hypoxia are well characterized at the level of individual cells (neurons and astrocytes) but, on their own, they cannot explain the effect observed on cognition at larger scales.

The investigation of brain networks can help characterize brain function at larger scales. In particular, resting state networks (RSN) have been used to investigate activation patterns that the brain exhibits during rest^25^. This approach assumes that neural networks remain active during rest, and that their correlated activity can be used to map the functional connections without the need to perform stimulations or involve the subject in a task.

RSN are thought to be important in cognition and consciousness, and are altered in neurological dysfunctions such as Parkinson’s disease^26^, Alzheimer’s disease^27^, schizophrenia^28^ and autism^29^, among others. RSNs are also altered during long term exposure to hypoxia^12,30,31^. Patients with obstructive sleep apnea, associated with mild intermittent hypoxia each night, exhibit changes in RSNs when compared to controls^32,33^, as do people residing at a higher altitude for a longer period of time^31^. Both conditions have been associated with a decrease in cognitive performance^31,34–36^.

Most of the literature addressing the impact of hypoxia on functional connectivity has been performed during long-term exposure to low oxygen levels or high altitude. A recent exception is a study performed on mice using photoacoustic tomography during resting state^37^. This approach relies on neurovascular coupling and observed a decrease of functional connections between homotopic cortical regions during acute exposure to hypoxia. However, to better understand what underlies cognitive function impairment, investigations with more direct measures of brain function are needed. While RSNs investigated with fMRI have been shown to be altered because of hypoxia, these are not direct neuronal measurements. In this work, we aim to investigate changes in neuronal RSNs and hemodynamic signals in the mouse brain as a result of acute, global hypoxia.

With the development of highly sensitive genetically encoded calcium indicators such as GCaMP^38^ it is now possible to monitor functional connections using resting state calcium activity^39,40^ in mice with high sensitivity, specificity and precision, while simultaneously imaging the hemodynamic signals with intrinsic optical imaging^41^. We exploited this approach and imaged mice at several hypoxia levels to investigate concomitant changes in large scale brain networks.

## Material and methods

### Animals

All procedures were approved by the Animal Care Committee of the Université de Montréal (Comité de Déontologie en Expérimentation Animale, CDEA) and conformed to the Canadian Council on Animal Care and Use guidelines. Reporting of the experiments is done in line with the ARRIVE (Animal Research: Reporting in Vivo Experiments) guidelines.

Eight C57/BL6 mice were used (4/4, male/female). Mice were 3–4 months of age at the first imaging session, and 4.5–6 months at the last. All mice had ad libitum access to food and water. Male and female mice formed two separate groups, with the female group preceding the male by 6 weeks.

No previous data was suitable to base statistics for sample size. However, based on results previous research^16,42,43^ we estimated the needed sample size at 8.

The set-up of this study allowed normoxia and hypoxia comparisons within the same animal, and thus no control group was used. In this same line of reasoning, potential confounding factors such as difference in cage location between the groups were not controlled.

### Virus injection and window surgery

Mice were injected with AAV.PHPeB.syn.GCaMP6s.WPRE (Canadian Neurophotonics Platform – Viral Vector Core, Québec, Canada) in the tail vein at 8 weeks of age. In contrast to other serotypes, AAV.PHPeB capsides can cross the blood brain barrier and can be injected in the blood to infect neurons^44^. At least 5 days passed between injection and window surgery. To prepare the imaging window, mice were anesthetised with isoflurane (1.5–3%) and body temperature was maintained at 37 °C with a rectal probe and a heating pad. Heart rate and oxygen saturation were monitored during the surgery (Small animal physiological monitoring system, Labeo Technologies inc.). The head of the mouse was shaved followed by i.p. injection with an anti-inflammatory drug (Carprofen). A local analgesic (Lidocaine) was injected s.c. on the head and the skin was cleaned thoroughly with alcohol and iodine. The skin on top of the skull was cut away to expose the skull. A glass coverslip was secured over the exposed skull with clear dental cement (C&B Metabond)^45^. Cyanoacrylate (Vetbond) was used to bond the skin to the skull, and a titanium head bar was glued to the top of the head for fixation during imaging. After the surgery, the mice recovered for at least a week before the first habituation session (described below). The surgery was followed by three days of analgesia administration (Carprofen, 0.01 mL per gram).

One imaging window led to imaging artifacts 6 weeks after the start of imaging. The mouse was re-anesthetized, and a new window and head bar were installed with the methods described above. Following this procedure, the mouse followed the same imaging protocol as the other mice, with two weeks of delay.

### Mesoscale imaging

Imaging was done with awake mice, since anesthesia can impact hemodynamics and calcium resting state networks^46,47^. The mice were fixed by their head bar during imaging to avoid movements. To limit stress, mice were habituated to fixation and imaging over a period of a week, starting with 1 min on the first day and gradually increasing the duration depending on the behaviour of the mouse (grooming/eating behaviour shortly after release), until a period of 40 min was reached.

Imaging was performed with a LightTrack OiS200 system (Labeo Technologies inc.) and used a time-interlaced multicolor illumination. The GCaMP reporter was excited with a 475 nm centered blue LED (CREE XPEBBL-L1) in combination with an excitation filter (Semrock FF02-472/30-25). To capture the activity, a long-pass emission filter (Semrock FF01-496/LP-25) was positioned in front of the camera (sCMOS sensor) just after the imaging objective (50 mm F#1.2, Nikkor), which collected images at a framerate of 80 Hz. This yielded a final frame rate of 20 Hz per color. The collected images were 192 × 192 pixels over a 10 × 10 mm area of the brain, with an exposure time of 8.5 ms.

To disentangle the hemodynamic response from the calcium response, red, green and amber LEDs (OSRAM LZ4-20MA00, 620 nm, 535 nm, 590 nm respectively) were used to gather maps of absorption which were used to estimate the changes in concentrations of oxy- and deoxyhemoglobin.

To refrain whiskers from hovering above the brain and casting a shadow, we designed and 3D-printed a small cap that could be placed on the head and had an opening through which the skull could be seen and imaged. This hat also reduced the visual stimulation that the mouse received.

### Hypoxia protocol

**Figure 1:**
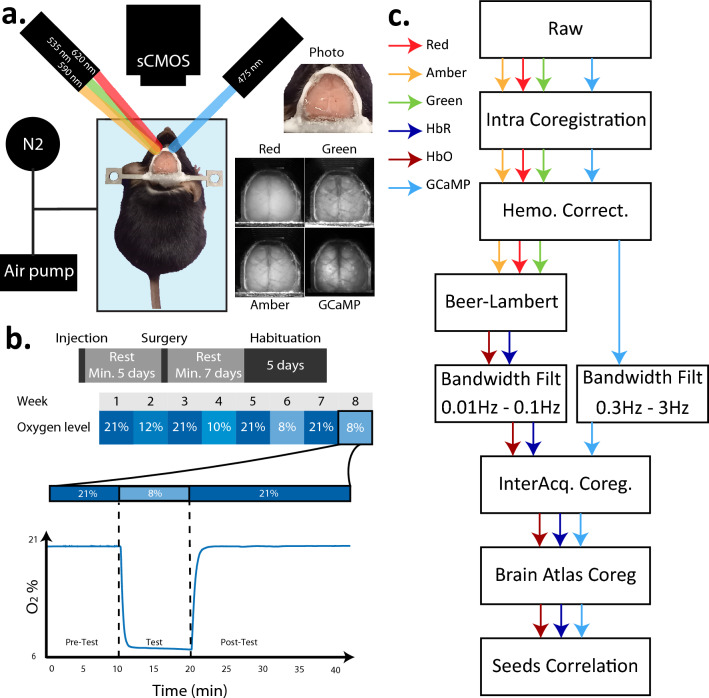
(**a**) Overview of the imaging set-up. The mouse is fixed by its head bar inside a container where the influx of oxygen is monitored. To excite the GCaMP molecules, blue light was used, while red, green and amber lights were used for hemodynamics calculations. (**b**) Overview of the hypoxia protocol. Upper panel: Mice were injected with a GCaMP virus, and after a minimum of 5 days rest underwent window surgery. After a week of rest, mice were habituated to the fixation and were subsequently imaged once a week, with intermittent hypoxia and normoxia conditions. Lower panel: example of oxygen concentration for an experiment of the impact of 8% hypoxia. Oxygen levels dropped quickly after onset of nitrogen influx. (**c**) Analysis pipeline of the imaging data.

The mouse was placed in a transparent, airtight box (19 cm × 26 cm × 7 cm) for which influxes of air and nitrogen were controlled (Fig. 1a). During normoxia, atmospheric air was pumped into the box (1L/min). To decrease oxygen levels, the influx of nitrogen into the box was initiated and increased until the desired oxygen level was reached. Inflow was active and achieved with a pump, outflow was passive through a small tube. Oxygen levels were calibrated before the start of each acquisition with the help of an oxygen sensor (LuminOx O2 Sensor—Lox-O2-S), so that the transition from normoxia to hypoxia during imaging was fast and accurate (Fig. 1b). Imaging was done through the transparent lid of the box.

Mice were imaged once a week. To monitor any chronic changes in resting state networks over time due to the hypoxia-manipulations, we interlaced sessions of normoxia between the hypoxia acquisitions. Thus, we started the first weekly session with normoxia, then 12% oxygen, normoxia, 10% oxygen, normoxia, 8% oxygen, normoxia, ending with another acquisition at 8% oxygen. Levels of 12, 10 and 8% oxygen correspond to partial pressure of O2 in atmosphere (pO2) at 4000, 5500, and 7000 m, respectively. Acquisitions lasted 40 min, starting with 10 min normoxia, followed by 10 min of the hypoxic condition (where applicable) and ending with 20 min normoxia (Fig. 1b). During acquisitions, the oxygen in the box was monitored with an oxygen sensor (LuminOx Optical Oxygen Sensors connected to an Arduino Uno).

Hypoxia was started and stopped by manually flipping a switch, which could have introduced a small imprecision in timing. To minimize this impact on the quantifications, the transition periods were excluded from the analysis.

Outcome measures of this experiment were the changes in hemodynamics, as defined by an increase or decrease in oxyhemoglobin (HbO), deoxyhemoglobin (HbR), total hemoglobin (HbT) and oxygen saturation levels (sO2), and changes in the neuronal connectivity, as defined by the increase or decrease in correlation in GCaMP activity between seeds during hypoxia compared to during normoxia, as well as over time in normoxia conditions.

### Exclusion criteria

A-priori exclusion criteria for this experiment were infection, illness, or death of the animal. Furthermore, animals that failed to express the GCaMP virus (n = 1) were excluded from the functional connectivity analysis (n = 7). This mouse remained included in the hemodynamic results (n = 8).

Several circumstances occurred during the study that resulted in partial exclusion of acquisitions. First, one mouse displayed clear divergent GCaMP signal in the last acquisition (suspected epileptic seizure or similar). Data from this event (at 30 min) and after were excluded from the analysis. Second, one acquisition of 12% oxygen showed technical errors, which resulted in cutting the data of that mouse after 35 min from the analysis. Third, at the 10% oxygen acquisition for the female batch (n = 4), the timing of the hypoxia onset occurred at 18 min instead of 10, so the first 8 min of the acquisition were discarded, and the last 8 min consisted of missing values. Lastly, the timing for returning to normoxia was off for one mouse at the 8% oxygen acquisition, which resulted in discarding the data after 30 min. None of these alterations affected the hypoxia period, nor the first normoxia period, and thus all statistics and results are concluded on a n = 7 for GCaMP data and n = 8 for hemodynamic data. No mice died during this study.

### Analysis

Analysis was done with MATLAB R2020b and followed a series of steps (Fig. 1c). Analysis was not done blindly, and the condition of the oxygen level at the acquisition was known at all times by the authors.

#### Co-registration

To correct for movement during the acquisition, every frame was co-registered to the first frame of the acquisition using an affine 2D transformation with the mean squared error as the optimisation parameter. Following that, a diffeomorphic transformation was computed to correct for movements or small deformations generated by the non-rigid nature of the mouse brain. With heartbeat and respiration, the vessels of the brain might have small local movements that would be better adjusted for in a non-rigid co-registration than a rigid one.

#### Hemodynamic correction

The fluorescence signal was corrected for hemodynamic fluctuations using reflectance measurements obtained from the red, green and yellow LEDs, as described in^48^. Shortly, the backscattering light of each one of these LEDs provides information about the variations in oxy- and deoxy- hemoglobin. Since each of the LEDs’ absorption spectrum is influenced differently by the two hemoglobin variations, we can use a linear regression of the reflectance signals to remove most of the hemodynamic fluctuations from the fluorescence signal. Doing so, most of the hemodynamic influence on the fluorescence measurement was corrected. The method was initially validated using GFP expressing mice where no fluorescence fluctuations were observed. Valley et al. showed in their recent work that this method works better than using the Beer-Lambert approximation of HbO and HbR. One reason could be that there are no a priori regarding baseline concentrations or pathlength factors in the computation. In order to get quantitative values of HbO and HbR, the Beer-Lambert approximation is still necessary, but in the case of hemodynamic correction it seems that a blind approach is more efficient.

#### Calculation of hemodynamic signals

The absorption spectra for HbO and HbR vary as a function of wavelength. This causes different reflectance for the red, green and amber lights. The same methods as described in^48^ were used, using the modified Beer-Lambert law to approximate the changes in HbO, HbR and HbT, assuming baseline values of HbO and HbR of 60 µM and 40 µM respectively^49–51^. To evaluate the impact of the different dynamics in HbO and HbR variations on the oxygen availability within the cortex, the cerebral oxygen saturation was calculated (sO2 = HbO/HbT).

#### Normalization

Normalization was applied on the fluorescence channel to obtain the percent fluorescence fluctuation (∆F/F) measurements. A band-pass filter of 0.3–3 Hz was used to obtain the signal of interest. The baseline fluorescence signal was obtained from a low pass filter at 0.3 Hz.

#### Co-registration between days

The Allen Mouse Common Coordinate Framework atlas^52^ was fitted on the first acquisition of each mouse using the bregma location. To ensure the consistency of the ROI placement, all acquisitions were co-registered to the first acquisition of that mouse, with the same method as described above.

#### Global signal regression

To underline the spatial changes caused by hypoxia, a global signal regression (GSR) was applied before the production of the seed pixel correlation maps of both the fluorescence and hemodynamic data^53^. Each pixel was normalized by the linear fit of the global signal (average of all pixels for every frame) on its own temporal dimension. Maps without GSR can be found in Supplementary 2. All quantitative and statistical analysis were done on data without GSR.

#### Regions of interest

Regions of interest (ROI) were identified with the Allen atlas^52^. For each mouse, an activity map was generated by summing the ∆F/F signal over the whole acquisition. The difference in baseline levels of activity of functional areas made it possible to position the atlas on the activity map. Regions of the atlas were pooled into right (R) and left (L) versions of Motor (Mot), Retrosplenial (Ret), Visual (Vis) and Somatosensory (Sen) regions (overview in Fig. 6C). For seed analyses, the pixel at the centroid of the region was taken and expanded with a 3-pixel radius disk. The time course of the average of these pixels was calculated and compared to other regions.

#### Statistics

For all statistical tests, p < 0.05 was deemed significant. If a correction for multiple testing was done (Benjamini–Hochberg False Discovery Rate—FDR^54^), corrected p-values are referred to as q-values, and similarly deemed significant if q < 0.05.

#### Hemodynamics

Values of HbO and HbR that differed from the mean with more than 3 standard deviations were considered outliers and excluded from the analysis. Mean HbO, HbR, HbT and sO2 values were calculated over the whole brain, with exclusion of the middle cerebral sinus and the edges of the brain.

For calculating dips/peaks in the dynamics of the hemodynamic data, the minimum/maximum value was found within a window around the observed dip/peak in the data, and the average and standard error of the mean (SEM) were calculated from that. The values were then compared to a normoxia period with the same time-window.

For the plateaus, the averages of minute 14.5 to minute 19.5 were calculated and compared to minutes 4.5 to 9.5 of the same acquisition. Since differences were not normally distributed (Kolmogorov–Smirnov test), a Wilcoxon-signed rank test was applied. To decrease the false discovery rate (FDR) due to the many statistical tests, a Benjamini–Hochberg FDR was applied^54^.

#### Correlation of the seeds

Both the GCaMP and HbO time courses of the ROI were extracted from the data. To evaluate the direct change that hypoxia causes, the correlation values of each unique seed pair before and during hypoxia were transformed with the Fisher z-transformation and then subtracted from one another. For the correlation matrices, the differences in correlation values were tested for normality with a Kolmogorov–Smirnov test, and were deemed not normally distributed, so a Wilcoxon signed rank test was chosen. Again, a Benjamini–Hochberg FDR was applied.

To compare the changes in connectivity within or between macroclusters, a Fisher z-transformation was performed, and the average change in correlation of seedpairs within the same macrocluster was calculated per mouse. The same was done for seedpairs between macroclusters. After verifying normal distribution and homogeneity of variance, t-tests were done for each hypoxia level. A Benjamini–Hochberg FDR was applied.

#### GCaMP normoxia levels

To see long-term changes over weeks, minute 2.5 to 7.5 (normoxia) of each acquisition was compared. A Friedman test was done for each unique seed pair. The seed pairs were corrected for multiple testing with a Benjamini–Hochberg FDR. If the Friedman test resulted in significant values, a Kruskal–Wallis test was done for each week compared to week 1.

## Results

### Hemodynamic response to hypoxia

#### Deoxygenated hemoglobin levels

**Figure 2:**
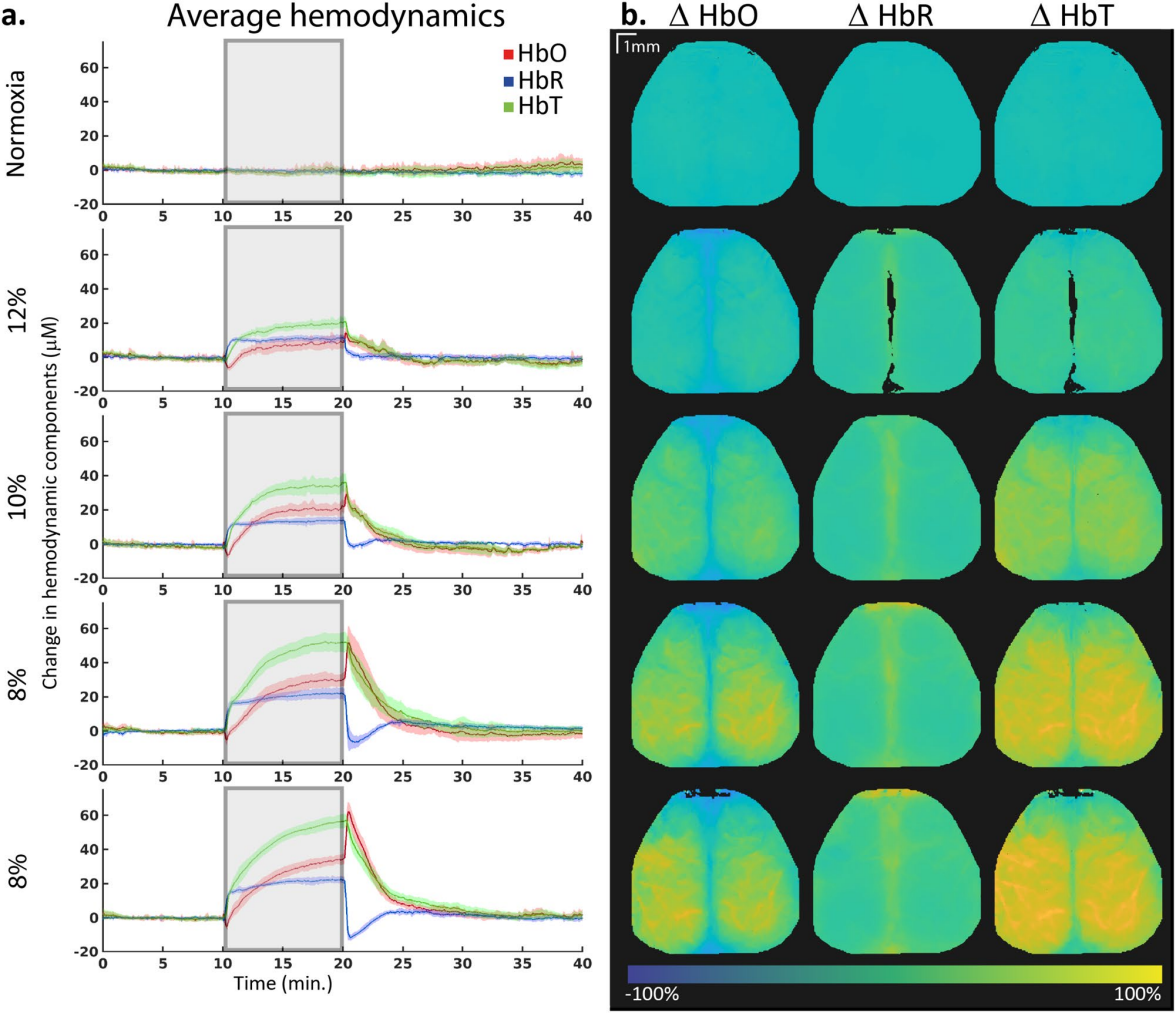
(**a**) Average changes in HbO, HbR and HbT concentrations over the whole brain. All mice are combined for these graphs (n = 8). The solid line indicates the mean, and the shaded regions indicate 95% confidence interval. Hypoxia started 10 min into the acquisition and was stopped at 20 min, as indicated with the grey area. (**b**) Spatial representation of changes in HbO, HbR and HbT concentrations during normoxia compared to the hypoxia period of the same acquisition (n = 1).

Within a minute after the start of the hypoxia period, HbR rapidly increased, and ∆HbR reached a plateau with a mean of 10.9 ± 0.5, 13.4 ± 0.4, 21.2 ± 0.7 and 21.6 ± 0.52 µM (mean ± SEM) for 12%, 10%, 8% and 8% oxygen, respectively (− 0.7 ± 0.5, − 1.0 ± 0.5, 0.3 ± 0.4 and − 0.1 ± 0.4 for normoxia conditions, Fig. 2a). All plateaus were significantly higher than baseline (q = 0.016 for all hypoxia conditions, q = 0.13, 0.44, 0.74 and 0.42 for the subsequent normoxia acquisitions, n = 8, Wilcoxon signed rank test, FDR corrected). Upon return to normoxia, we observed an undershoot which was more pronounced in acquisitions with lower oxygen levels.

#### Oxygenated hemoglobin levels

HbO shows a slower change in concentrations as a response to hypoxia compared to HbR (Fig. 2a). At the start of hypoxia, ∆HbO levels dipped briefly before increasing and reaching a plateau. Whereas ∆HbR plateaus were generally reached within a minute after hypoxia onset, plateaus for ∆HbO were reached later, and more severe levels of hypoxia delayed the response. At 12% oxygen, ∆HbO levels reached a plateau around 4 min after the start of hypoxia (7.8 ± 1.1 µM), while for 10% oxygen this was after approximately 6 min (20.0 ± 0.8 µM). ∆HbO levels in 8% oxygen did not reach a plateau within the hypoxic duration of 10 min, but the averages over 14.5 to 19.5 min were 28.0 ± 1.9 and 30.2 ± 2.6 µM, respectively (− 0.2 ± 1.2, − 0.9 ± 0.8, − 2.8 ± 0.7, and − 0.8 ± 0.7 for normoxia acquisitions). All plateaus differed significantly from baseline (q = 0.016 for all hypoxia conditions, q = 1, 1, 0.24 and 0.73 for the subsequent normoxia acquisitions, n = 8, Wilcoxon signed rank test, FDR corrected).

Upon return to normoxia, ∆HbO showed a quick overshoot which intensified with lower levels of oxygen, with maxima of 14.3 ± 2.4, 29.0 ± 6.4, 51.8 ± 13.8 and 62.1 ± 6.9 µM for 12%, 10%, 8% and 8% respectively, calculated by averaging the maximum value of each mouse between 20 and 21 min. The maxima for normoxia acquisitions were 5.1 ± 5.4, 3.5 ± 3.5, 5.0 ± 2.8 and 3.9 ± 6.2.

#### Spatial distribution

**Figure 3:**
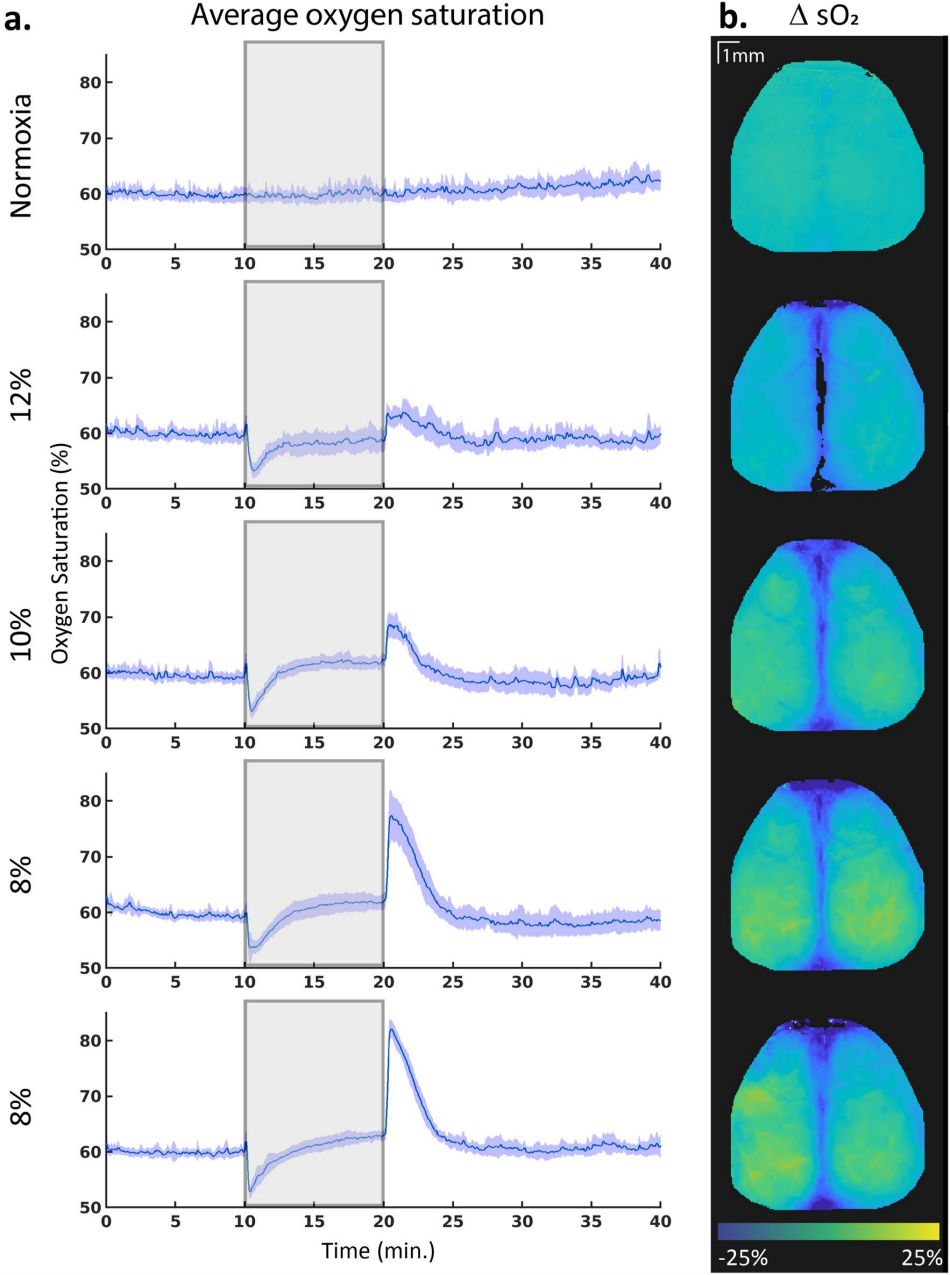
(**a**) Average changes in oxygen saturation over the whole brain. All mice are combined for these graphs (n = 8). The solid line indicates the mean, and the shaded regions indicate 95% confidence interval. Hypoxia started 10 min into the acquisition and was stopped at 20 min, as indicated with the grey area. (**b**) Spatial representation of changes in oxygen saturation (sO2) concentrations during normoxia compared to the hypoxia period of the same acquisition (n = 1).

The increase in HbO and HbR was not localized to a single brain region, but rather showed a similar pattern throughout the brain (Fig. 2b). HbO was slightly more augmented in the tissue regions, while HbR showed more increase in the middle cerebral vein. This same spatial pattern was visible in the sO2 increase (Fig. 3b), where the increase was found in tissue and not the middle cerebral vein, with no preference for a specific brain area.

#### Oxygen saturation

sO2 levels first dropped rapidly, dipping to consistent levels over different hypoxia levels from 60 ± 1.5, 59 ± 0.9, 59 ± 0.4 and 60 ± 0.9% during baseline (59 ± 0.6, 59 ± 0.8, 59 ± 0.5, and 59 ± 0.6 in normoxia conditions) to 54 ± 1.0, 54 ± 1.5, 54 ± 2.3 and 54 ± 1.5% for 12%, 10%, 8% and 8% oxygen, respectively (59.8 ± 1.0, 59.6 ± 1.1, 58.0.5 ± 0.4 and 59.3 ± 0.6 for normoxia acquisitions). This dip was reached within half a minute after the start of hypoxia and was significant compared to the baseline (q = 0.013, for all hypoxia conditions, q = 0.63, 0.51, 0.01 and 1 for the subsequent normoxia acquisitions, n = 8, Wilcoxon signed rank test, FDR corrected, Fig. 3a).

After this initial dip, sO2 levels slowly climbed back to baseline, and reached a plateau. Plateaus were reached later at lower levels of oxygen. Surprisingly, this saturation was slightly higher than the baseline for the 10% and 8% hypoxia conditions (plateaus 59 ± 0.6, 62 ± 0.3, 62 ± 0.4 and 62 ± 0.6%, baselines 60 ± 0.6, 59 ± 0.5, 59 ± 0.4 and 60 ± 0.4%). However, this difference was not significant in all hypoxia levels (q = 0.33, 0.03, 0.15 and 0.03 for 12%, 10%, 8% and 8% oxygen, respectively, n = 8, Wilcoxon signed rank test, FDR corrected), and the normoxia acquisitions showed similar values (baseline: 60 ± 0.5, 60 ± 0.5, 59 ± 0.6 and 60 ± 0,5%, plateau: 60 ± 0.7, 60 ± 0.5, 58 ± 0.5 and 60 ± 0.5%, q = 0.64, 0.53, 0.16 and 0.33).

Upon return to normoxia, an overshoot could be observed that was stronger with lower oxygen levels (64 ± 2.7, 69 ± 2.6, 77 ± 6.0 and 82 ± 2.4% for 12%, 10%, 8% and 8% of oxygen, respectively, 63 ± 2.5, 62 ± 3.4, 63 ± 1.7, and 62 ± 2.8 for normoxia acquisitions). Peaks at 10% and 8% oxygen differed significantly from baseline, whereas the peak at 12% did not (q = 0.13, 0.02, 0.02 and 0.02 for 12%, 10%, 8% and 8% oxygen, respectively, q = 0.74, 0.33, 0.02 and 0.36 for the subsequent normoxia acquisitions, n = 8, Wilcoxon signed rank test, FDR corrected).

### Neuronal activity

#### Activation over time

Analysing activation directly is not possible with the normalization and filtering that is used in the rest of the analysis. The analysis was redone without normalization in order to depict the activation (Supplementary 1). For consistency, we will focus on the fluctuations in the data that is following the main pipeline.

**Figure 4:**
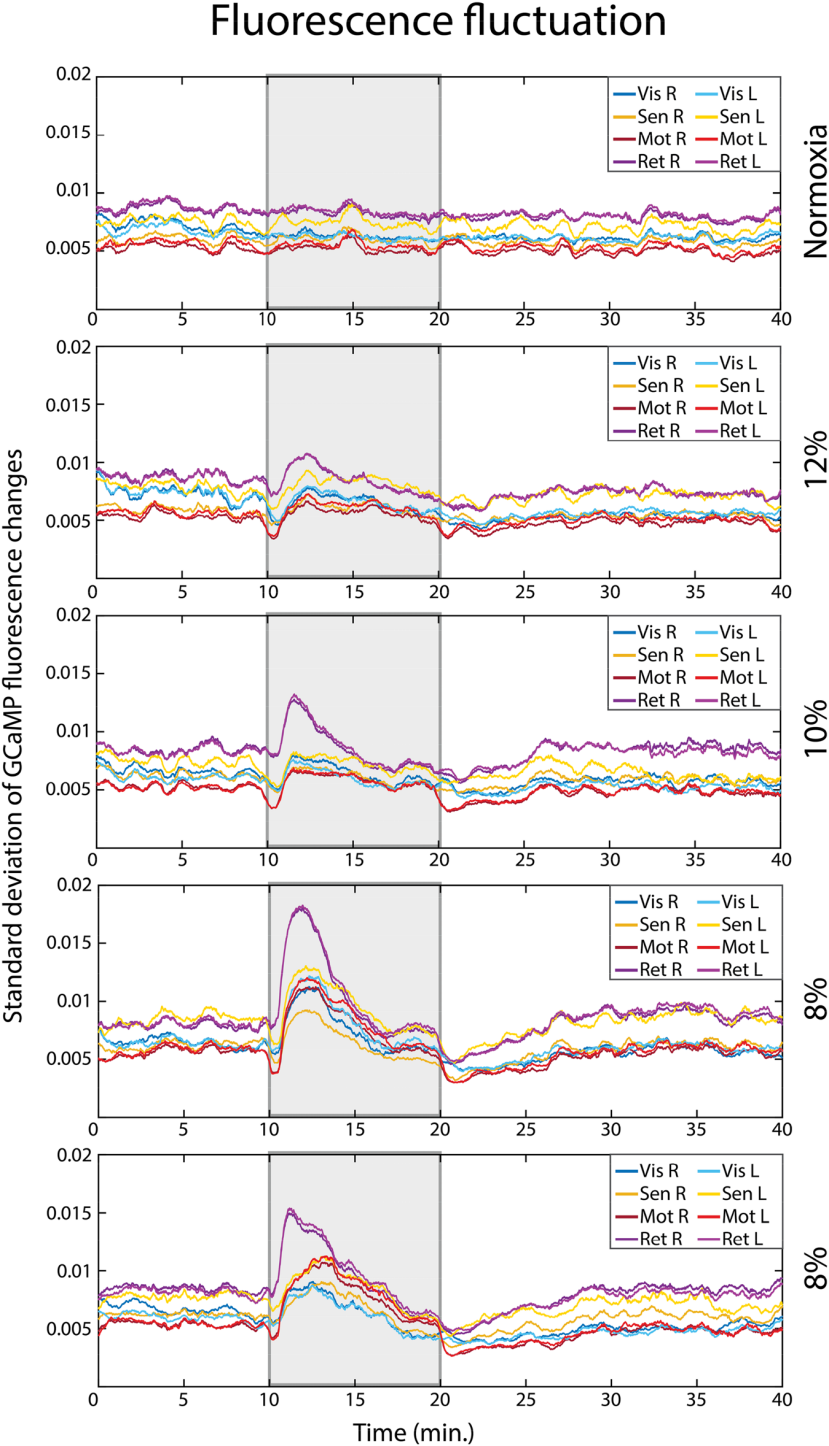
Standard deviation over time, depicted per seed, averaged over all mice (n = 7). The standard deviation was calculated with a window of 10 frames. Grey areas indicate the timing of the hypoxia period.

For each seed, the fluctuations of the fluorescence caused by GCaMP were quantified using the standard deviation (std) within a moving window of 10 frames (500 ms, Fig. 4). Upon the onset of the hypoxia period, we observed a transient decrease in std, followed by a large increase. The transient decrease mimicked the changes seen in HbO at the onset of hypoxia (Fig. 2). However, unlike the HbO levels, the increased fluctuations of GCaMP fluorescence did not stay consistent over the hypoxia period, but rather dropped after an initial peak. Independent of the level of hypoxia, the greatest increase in std were observed in both the right and left retrosplenial seed (shown in purple, Fig. 4).

#### Seed pixel correlation maps

**Figure 5:**
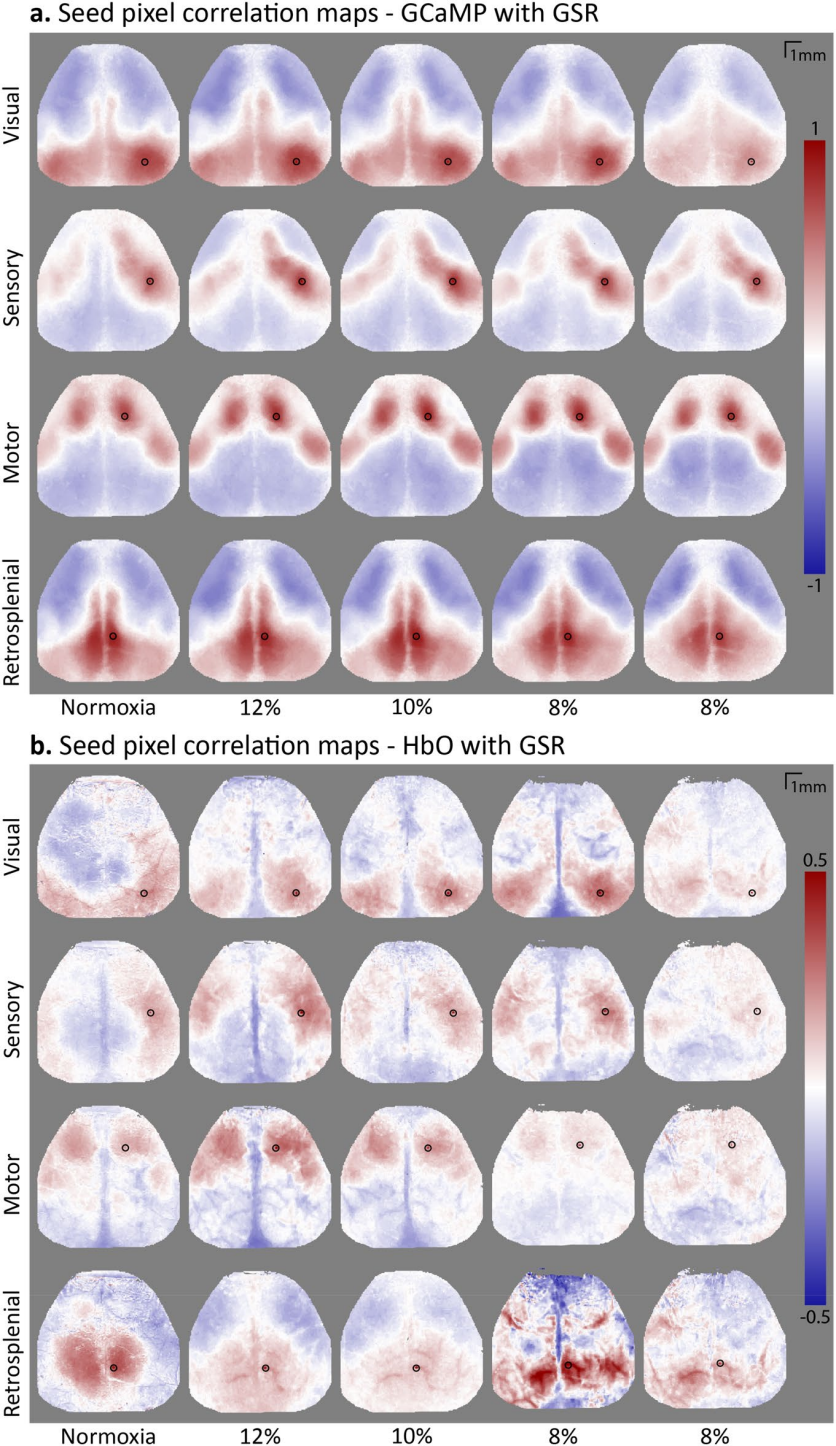
Seed pixel correlation maps (SPCM) over minute 12.5 to 17.5 of the first normoxia, and all hypoxia periods (n = 1). A global signal regression (GSR) was applied (see Supplementary 2 for maps without GSR). The black circle indicates the chosen seed; the centroid of the region of interest. Red indicates positive correlation of the seeds timecourse to the corresponding pixel, blue indicates negative correlation. (**a**) SPCM of fluorescence caused by GCaMP, indicating neuronal activation. (**b**) SPCM of HbO concentrations, measured with intrinsic optical imaging.

To explore the impact of hypoxia on the functional connectivity, the cortical fluctuation in centroids of the different ROI were correlated with the fluctuation in every other pixel over the cortex (Fig. 5a, Supplementary 2 for maps without GSR).

As expected, these results showed strong connectivity between homotopic regions, particularly between the two visual, motor and retrosplenial cortices. At the intrahemispheric level, a strong reciprocal connection existing between the motor and somatosensory cortex was also observed. Finally, weak and even negative connections could be observed between the anterior (including motor and somatosensory cortex) and posterior (including visual and retrosplenial cortex) regions, both inter- and intrahemispherically, supporting the idea that these groups of regions are part of two independent functional clusters (hereafter referred to as macroclusters). This is also supported by the strong similarity of the correlation maps of motor and somatosensory seeds on the one hand, and visual and retrosplenial seeds on the other hand.

When comparing the seed pixel correlation maps based on GCaMP signals to the ones based on HbO fluctuations (Fig. 5b), the resemblance was clear. The connections within anterior and posterior structures were present in both imaging methods, albeit less clearly in the hemodynamic maps. These similarities were expected since neurovascular coupling dictates that an increase in neuronal activation is accompanied by a local increase in HbO.

#### Changes in connectivity during hypoxia

**Figure 6:**
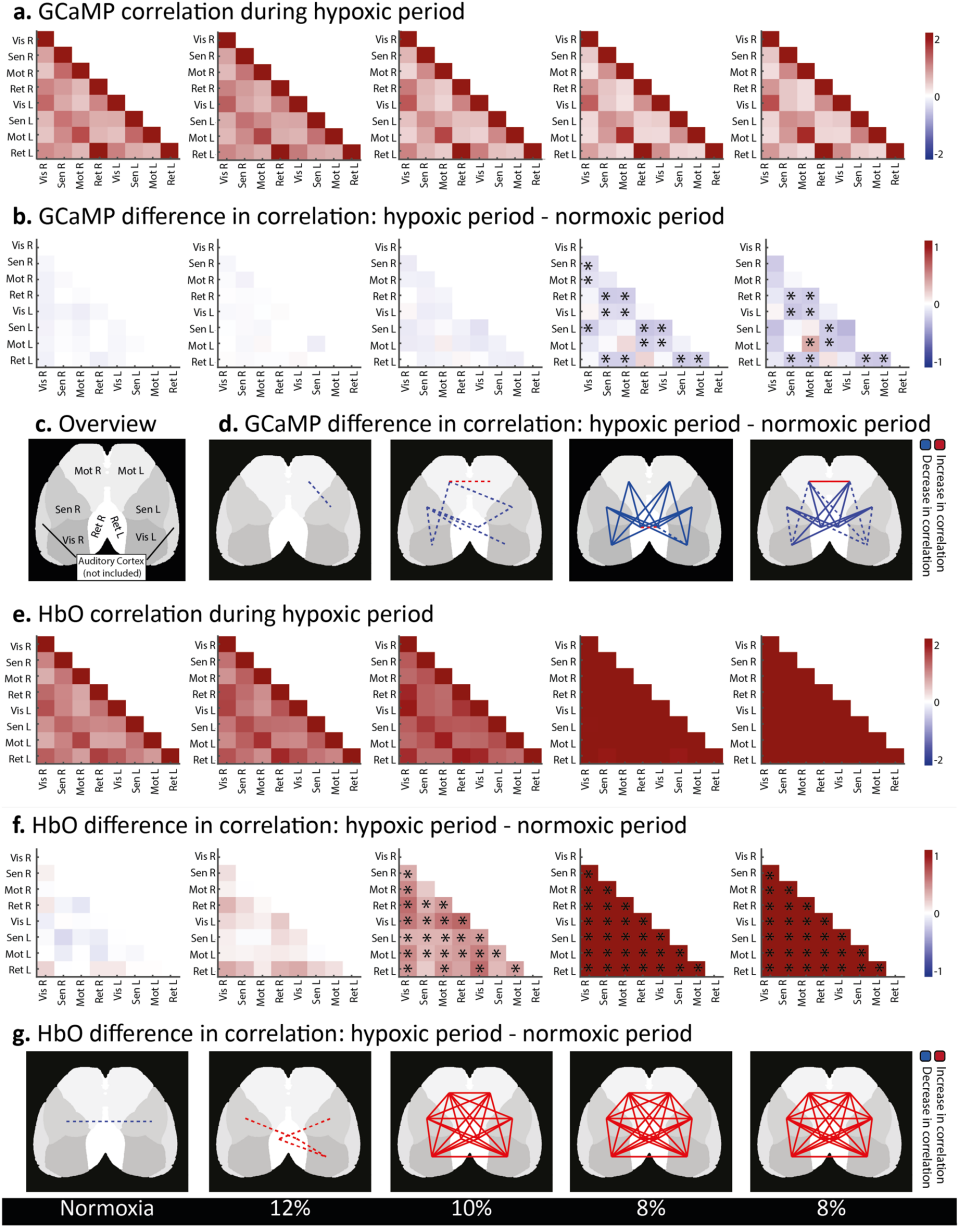
Correlation during hypoxia, and changes in correlation over different hypoxia levels, from normoxia (left column) to 8% oxygen (right column). (**a**) Correlation matrices depicting the correlation of the seeds based on the amount of fluorescence caused by GCaMP, during the hypoxia period (minute 12.5 to 17.5) (n = 7). (**b**) Differences in fluorescence correlation of the seeds during hypoxia compared to the normoxia (minute 2.5 to 7.5) period of the same acquisition (n = 7). Asterisks indicate q < 0.05. (**c**) Overview map indicating the regions used for this analysis. (**d**) The difference in correlation of the seeds depicted in B shown on the regions of interest. Changes of the first normoxia acquisition are not shown. Dotted lines indicate p < 0.05 before false discovery rate (FDR) adjustment, filled lines indicate q < 0.05 after FDR adjustment. Colours indicate an increase (red) or decrease (blue) in connectivity during hypoxia compared to the normoxia period before. (**e**) Correlation matrices depicting the correlation of the seeds based on the amount of HbO during the hypoxia period (n = 8). (**f**) Differences in HbO correlation of the seeds during hypoxia compared to the normoxia period of the same acquisition (n = 8). Asterisks indicate q < 0.05. (**g**) The difference in correlation of the seeds depicted in F shown on the regions of interest. Dotted lines indicate p < 0.05 before FDR adjustment, filled lines indicate q < 0.05 after FDR adjustment. Colours indicate an increase (red) or decrease (blue) in connectivity during hypoxia compared to the normoxia period before.

The correlation between the seeds was generally high in normoxic conditions, both when measured with GCaMP (Fig. 6a) as with HbO (Fig. 6e). As the oxygen levels dropped, we see a general decrease in connectivity based on GCaMP fluorescence (Fig. 6a), whereas the connectivity based on HbO signal increased in all seeds (Fig. 6e). This is seen more clearly when subtracting the correlation during normoxia from the correlation during hypoxia, within the same acquisition (Fig. 6b,f). Both 8% oxygen acquisitions showed a significant increase in HbO correlation for all seed pairs (all q-values were 0.0078, Fig. 6f,g). However, this increase is most likely due to a general increase in blood flow (as shown in Fig. 2), and thus does not inform us about the relationships between the seeds to the same extent as the GCaMP data.

Using the GCaMP data, significant differences in correlation values were found in the two 8% oxygen acquisitions, although a similar pattern could be seen emerging at the 10% oxygen acquisition as well (Fig. 6b,d). Decreases in connectivity were mainly observed between the anterior (motor and somatosensory areas) and posterior (visual and retrosplenial areas) macroclusters, with many interregional seed pairs significantly decreasing their correlation during hypoxia (Fig. 6b,d, see Supplementary 3 for all p-values of the 8% oxygen acquisitions). Contrarily, correlation between homotopic seed pairs or seed pairs within the same macrocluster (anterior or posterior) stayed relatively stable, and the connectivity between the homotopic motor seeds even significantly increased during the second 8% hypoxia acquisition.

#### Seed pairs within or between areas

**Figure 7:**
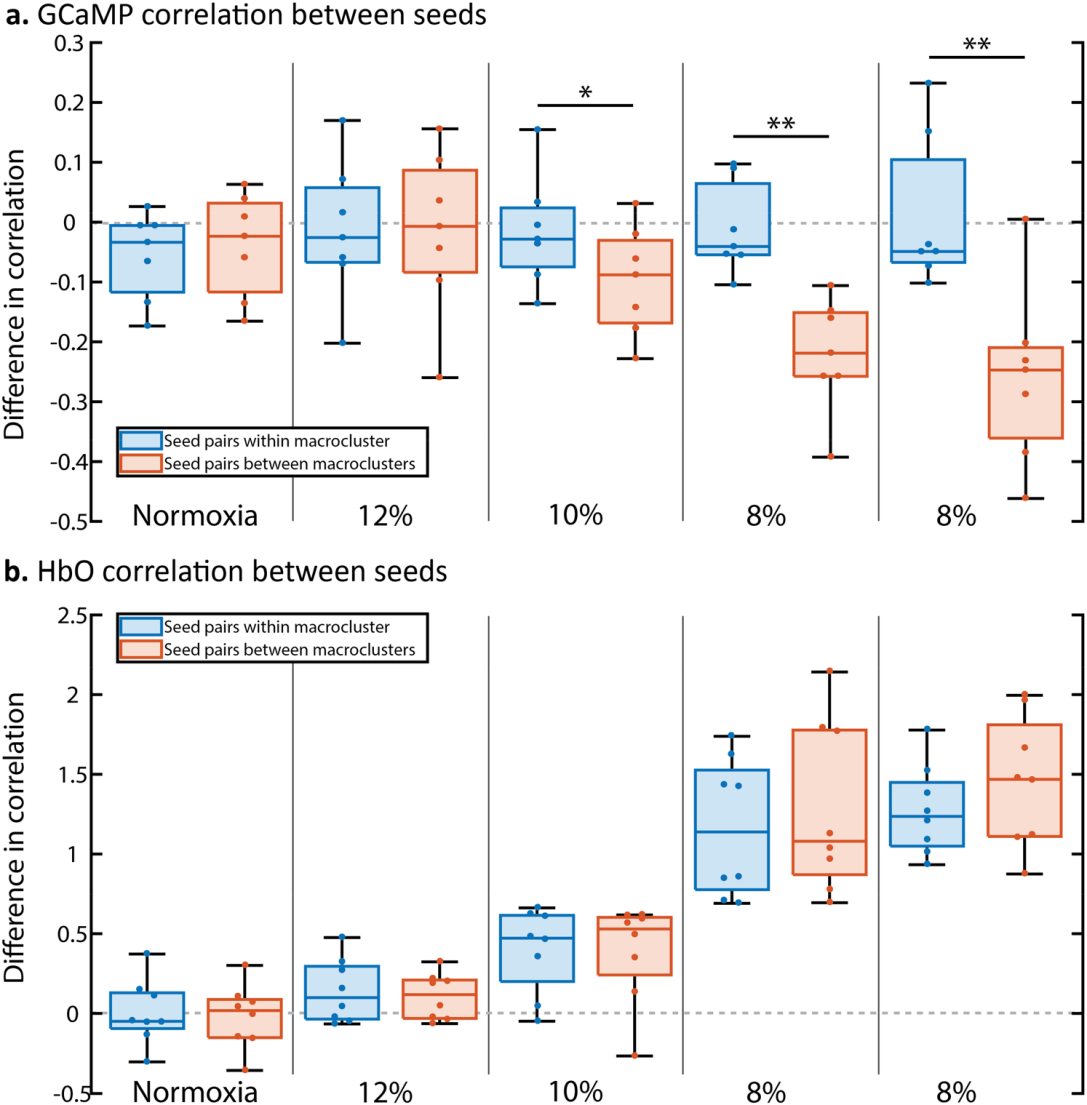
Differences in correlation of the seeds during hypoxia (minute 12.5 to 17.5) compared to the normoxia (minute 2.5 to 7.5) period of the same acquisition (n = 7), divided in seed pairs within the same area (both seeds in anterior, or both seeds in posterior area, orange boxplots) versus changes in seed pairs with seeds coming from different areas (blue boxplots). Each datapoint corresponds to the average change in correlation for all seedpairs falling into the abovementioned groups of one mouse. Edges of the boxes indicate the upper and lower quartiles, the line within the box indicates the median value. Whiskers extend to the furthest datapoints that are not considered as outliers. * indicates a q < 0.05, ** indicates q < 0.01 (t-test, false discovery rate (FDR) corrected). (**a**) Differences in correlation based on fluorescent (GCaMP) data, indicating neuronal activation. (**b**) Differences in correlation based on HbO concentrations, as measured with intrinsic optical imaging.

We further investigated the specific effect of hypoxia on connectivity between seeds within or between macroclusters (Fig. 7). The changes of connectivity differed significantly between “within” and “between” groups for the 8% acquisitions for the GCaMP (q = 0.0022 and q = 0.0022) but not the HbO (q = 0.3784 and q = 0.1031) measurements. GCaMP measurements also showed a significant difference for the 10% oxygen acquisition (q = 0.0448), whereas HbO did not (q = 0.7996). The groups did not differ significantly during the 12% oxygen acquisition (GCaMP: q = 0.9090, HbO: q = 0.4639) nor during normoxia (GCaMP q = 0.3510, HbO: q = 0.4639).

In conclusion, the GCaMP data suggests that the functional connectivity within macroclusters was relatively spared by hypoxia. On the other hand, seed pairs connecting different macroclusters showed a decrease in connectivity during hypoxia, with stronger decreases at lower oxygen levels, suggesting a decrease in communication between the anterior and posterior areas. Interestingly, the HbO data shows an opposite trend, with seed pairs connecting macroclusters increasing their correlation more than seed pairs within the same cluster.

#### Normoxia acquisitions

**Figure 8:**
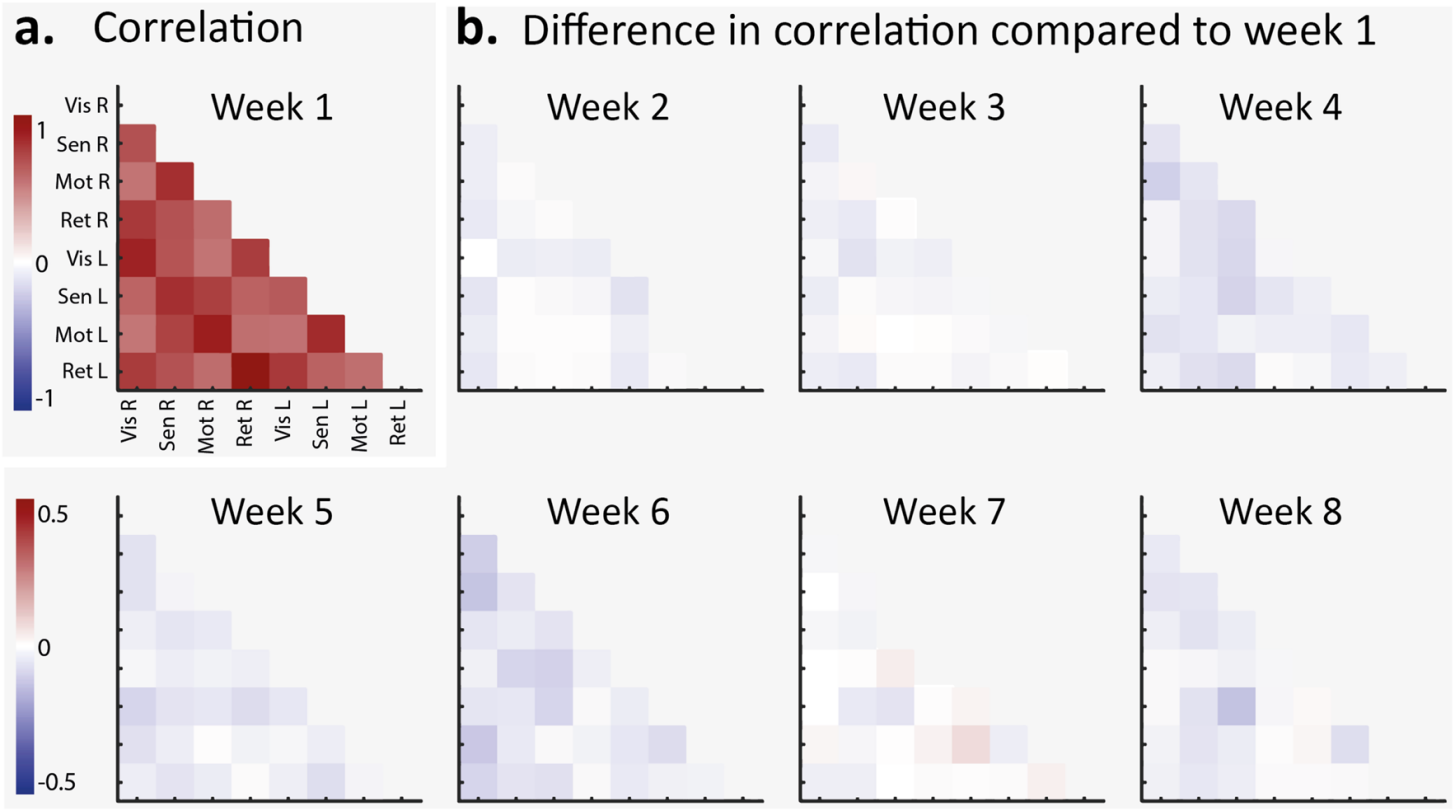
Comparisons of normoxia acquisitions. (**a**) Average correlation matrix for minute 2.5 to 7.5 of the first week (normoxia) for all mice (n = 7). (**b**) Difference in correlation between the normoxia period of that week (minute 2.5 to 7.5) and the normoxia period of week 1 (n = 7).

To control whether repeated exposure to hypoxia could have a longitudinal effect on connectivity states during normoxia, we depicted the connectivity of the first acquisition during baseline from minute 2.5 to minute 7.5 (Fig. 8a), and calculated the change in correlation of all subsequent acquisitions compared to the first (Fig. 8b). No significant changes were observed over time, suggesting that repeated exposure of hypoxia did not have a long-term impact on the connectivity in our animals.

## Discussion

### Hemodynamics

#### Increase in HbT

Hypoxia led to global brain hemodynamic changes that increased both HbO and HbR. The paradoxical increase in HbO during hypoxia can be explained by a general increase of cerebral blood flow (CBF) and cerebral blood volume (CBV), which both have been found before in hypoxic conditions^7–10,55^ and are thought to be a result of the prioritization of the brain over other organs^3^. For example, in conditions of 10% oxygen, CBF increases were about 15% in humans^9,11^. Spatially, this increase in CBF was not homogeneous over the brain^11,12^. However, in the present experiment, we did not observe any difference in hemodynamics between the imaged cortical regions when evaluating the complete hypoxia period (Fig. 2), which might be explained by our relatively small sample size. Similarly, the study of Julien-Dolbec et al.^55^ did not find a difference in CBV increase between brain regions, but rather a global increase during hypoxic periods. Further investigations measuring the spatial cerebral blood flow in parallel such as speckle contrast imaging^49^ should help to resolve this discrepancy.

#### Differences in increases of HbO and HbR

The HbO levels increased by a larger amount compared to HbR. This suggest a similar arterial response as is seen during neuronal activation, since an increase in blood flow will supply oxygenated arterial blood which, despite hypoxia, has higher saturation than what is measured in the capillary bed. Following an initial overshoot, return to the same level of oxygen saturation during hypoxia as during baseline suggests physiological oxygen regulation through a feedback mechanism.

Differences were also observed in the temporal profile of hemodynamic signals. Where the HbR reach their plateau quite quickly, HbO levels increase with a slower pace. The quick increase in HbR is likely due to the brain consuming the same amount of oxygen, while this is no longer being delivered by the arteries to the same extent, since the atmospheric oxygen level is lower. HbO levels increase more slowly, likely because the compensation mechanisms such as a change in respiration rate (depending on oxygen levels, shown in literature but not measured in this study^12,15^) are set into motion by feedback processes and need time to come into effect.

#### sO2 levels returning to baseline during hypoxia

The current study shows that the sO2 in the brain reaches the same levels during hypoxia as before the hypoxic episode. However, in the literature, oxygen saturation levels in blood have been found to be lower during mild hypoxic periods^8,9,16–18^. In humans exposed to an oxygen level of 10%, the sO2 levels decreased from 98 to 83%^9,18^ even though the oxygen consumption in the brain has been reported to remain the same^8,10,13,14,56^. However, these studies have mostly measured peripheral oxygen saturation levels^6,8,17,18^, whereas our study investigated cerebral sO2. Thus, a more comparable study would be that of Feng et al.^16^, who also measured oxygen saturation in the brain, yet also found a decrease in sO2. However, the time scale of their study was different from ours, with a hypoxia exposure of only 3 min. Nevertheless, even though the sO2 in our study dips right after the onset of hypoxia, the levels are already climbing back to baseline within 3 min of hypoxia exposure, whereas Feng et al. show continuous low levels of oxygen saturation after the induction of 10% oxygen. This difference could be partially explained by the use of isoflurane anesthesia during their recording, which is in contrast to the present results collected in awake mice^57^. Further research into this topic would be needed to understand the different results for this parameter, especially given the relatively small sample size of both the current study and the study of Feng et al. (n = 10).

### Neuronal activity

Although the hemodynamic results seem to implicate that there are sufficient compensatory mechanisms for oxygen delivery in the brain, we still observed a difference in neuronal connectivity as measured with GCaMP imaging. During the initial decrease of oxygenation at the start of hypoxia, the fluctuations in GCaMP increased, suggesting an increase in neuronal activation (also verified in Supplementary 1). These transient increases of GCaMP fluctuations showed a similar but slower pattern compared to oxygen saturation changes, with std values returning to baseline after about 5 min.

An increase in neuronal activation during an episode of decreased oxygenation seems paradoxical. However, this study is not the first to report such results. Chronic hypoxia has been linked to seizures^58^. Intermittent hypoxia has been shown to increase muscle sympathetic nerve activity^59^ and motoneuronal output^60^, and caused hyperexcitability in pyramidal neurons and interneurons in the hippocampus^61^. Thus, the origin of the transient increases of calcium fluctuations observed in the current study could have been attributed the an increase of cortical excitability during hypoxic episodes.

### Connectivity

Connectivity between seeds was quantified with both HbO and GCaMP measures. In normoxic conditions, the correlation matrix of these two measurements were very much alike, as expected^62^. As oxygen levels decreased, the connectivity between all seeds as measured with HbO increased. This is likely because of the increase in general blood volume in the brain. Since all seeds show an elevated HbO level, their correlation increases, with as a result that the correlation between seeds were all very close to 1 in the 8% acquisitions. This high correlation value also means that the increases measured in HbO seedpair correlation might be truncated, with naturally highly correlated seedpairs unable to show the same increase as seedpairs that were correlated to a lesser extent during normoxia.

On the other hand, correlations of seedpairs as measured with GCaMP showed opposite results. Changes consisted of decreases in connectivity, specifically between the anterior (including motor and somatosensory cortex) and posterior cluster (including visual and retrosplenial cortex) macroclusters. Changes were gradual between sessions as O2 decreased, suggesting that they indeed originate from the hypoxic condition. The changes in mild hypoxia of 12% and 10% oxygen were small, but at 8% oxygen we observed strong decreases in functional connectivity between the anterior and the posterior macroclusters. Since homotopic connections between seeds stayed relatively stable, this cannot be considered as a decrease in long range connectivity. Rather, it suggests a reduction in the interaction between modules operating distinct functions such as vision and navigation on the one hand (involving the visual and retrosplenial cortex) and sensorimotor functions on the other hand (involving the somatosensory and motor cortex)^39^. The observed increase of interhemispheric functional connections between the left and right motor seed in the last acquisition further reinforces the idea that the cortex is becoming more modular during hypoxia.

## Conclusion

In this work we investigated the hemodynamic and neural changes in resting networks associated with episodic hypoxia. We observed that despite the presence of physiological compensation to maintain oxygen saturation in the brain, neural resting state networks were modified during hypoxia, bringing about a decrease in connectivity between anterior and posterior brain areas, suggesting that measures of vascular physiology alone are not sufficient to characterize brain function during hypoxia.

### Supplementary Information

Supplementary Information.

## Data Availability

The code used for the analysis is available at https://github.com/marl1bakker/Hypoxia. Data is available upon request to the corresponding author.
