# Supplementary material for: Alteration of functional connectivity despite preserved cerebral oxygenation during acute hypoxia
Source: Sci Rep. 2023 Aug 15;13:13269. doi: 10.1038/s41598-023-40321-3 (PMC10427674; doi:10.1038/s41598-023-40321-3)
Supplement: Supplementary file 1 — Supplementary Information. [file 41598_2023_40321_MOESM1_ESM.docx]

Alteration of Functional Connectivity Despite Preserved Cerebral Oxygenation During Acute Hypoxia

Marleen E. Bakker^1,2^ *, Ismaël Djerourou^1^, Samuel Belanger^3^, Frédéric Lesage^2,4^, Matthieu P. Vanni^1^

**Supplementary 1.1**

***Methods activation seed pairs***


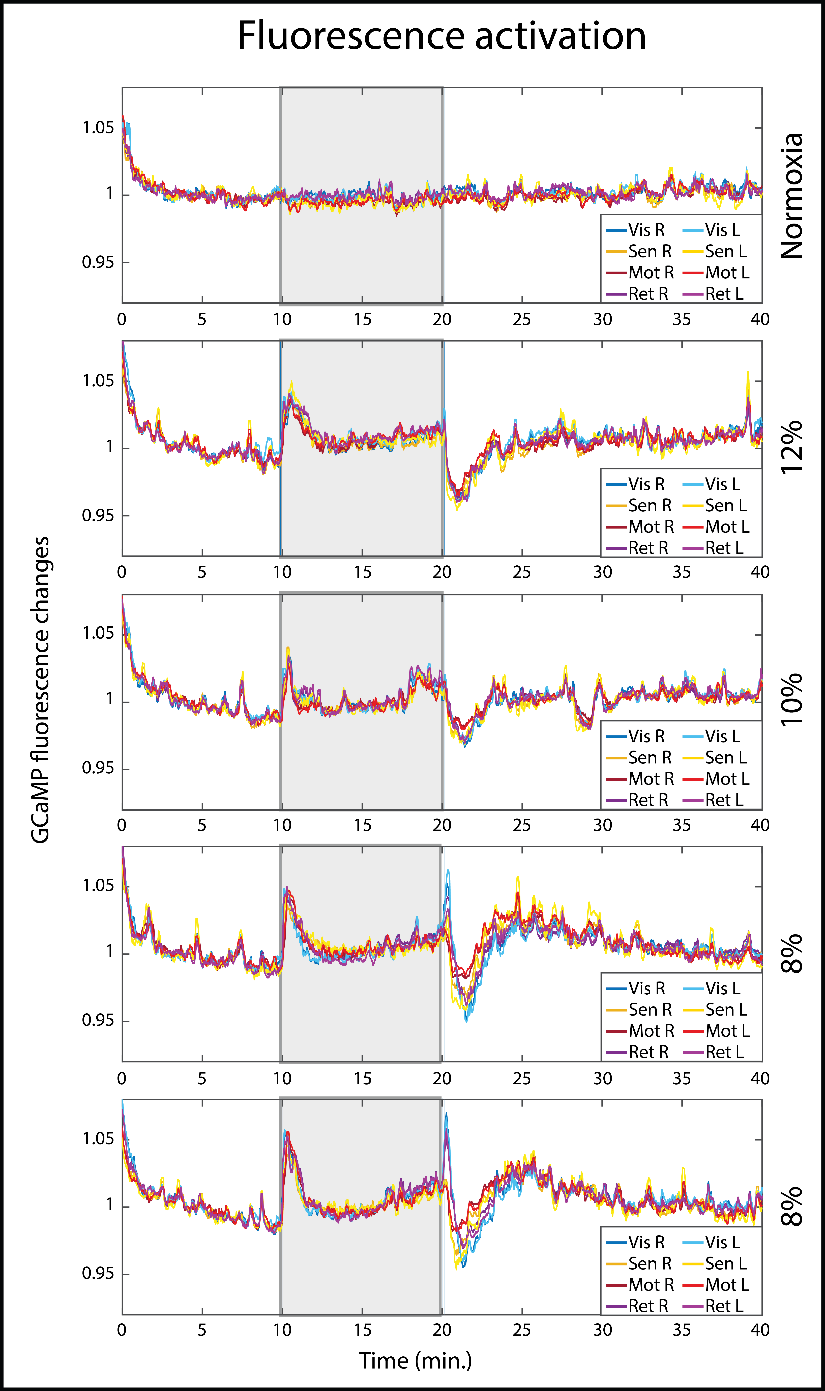
To be able to look at the activation of the seeds and to compare the amount of activation over time, the timecourses of the seeds were recalculated without normalisation and GSR. This means the images were co-registered and corrected for hemodynamics, and the timecourses were taken from the centroids of the regions of interests. Mice had a different level of fluorescence expression, either because of the succes rate of the virus injection or because of biological differences. This resulted in different amounts of fluorescence expression between mice. In order to compare between mice, we calculated the mean of minutes 2.5-7.5 of each seed for each mouse, and divided the whole timecourse by that number. Then, the mean over mice for each seed was taken and plotted (Supplementary 1.2). The steep decline at the start of the acquisition is caused by the exponential decay from the illumination source warming up.

**Supplementary 1.2**

Supplementary 1.2. Fluorescence activation over time, depicted per seed, averaged over all mice (n=7). Grey areas indicate the timing of the hypoxia period.

**
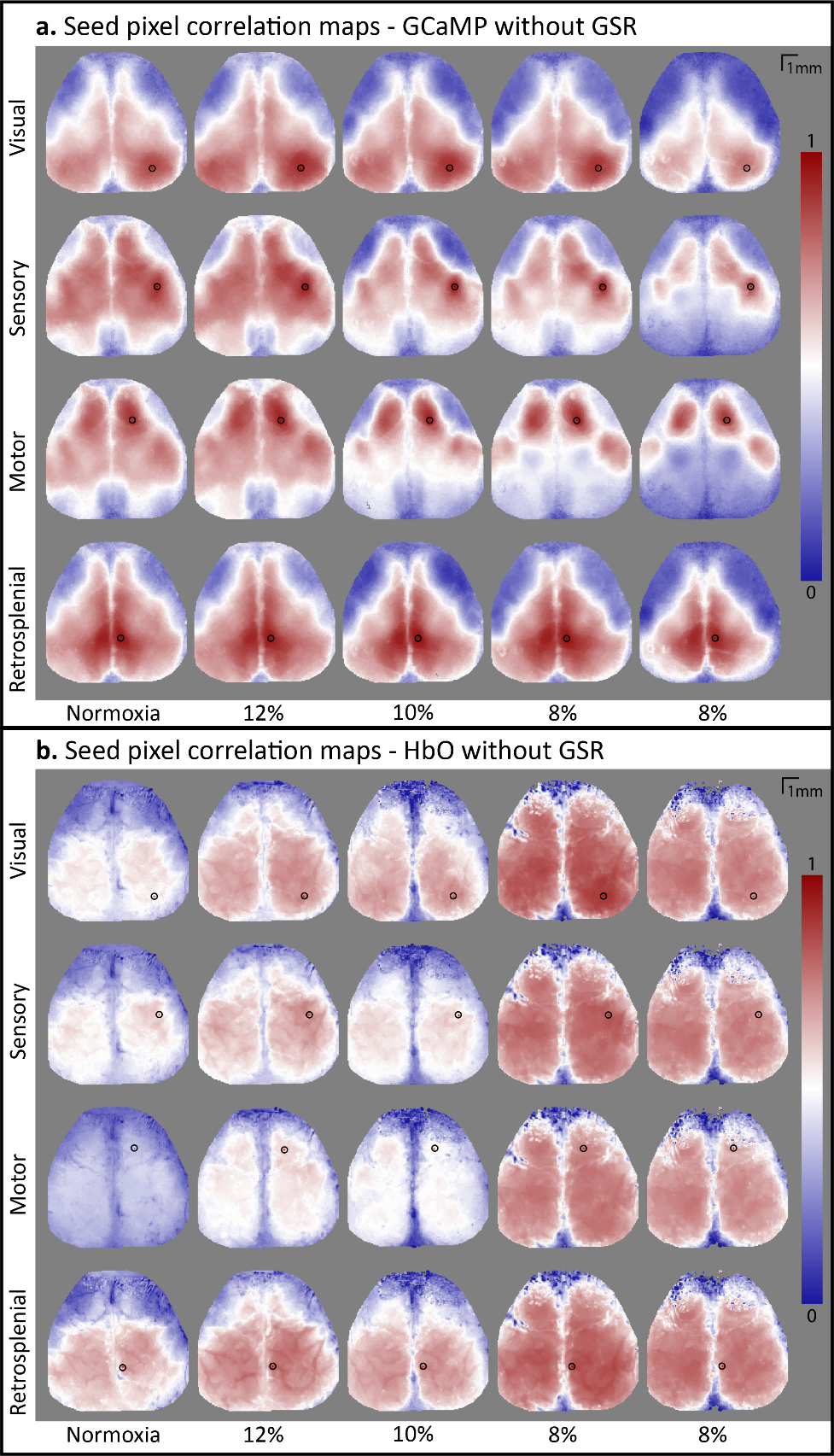
Supplementary 2**

Supplementary 2. Seed pixel correlation maps without GSR. Maps represent correlation over minute 12.5 to 17.5 of the first normoxia, and all hypoxia periods (n=1). The black circle indicates the chosen seed; the centroid of the region of interest. Red indicates positive correlation of the seeds timecourse to the corresponding pixel, blue indicates negative correlation. a. Maps based on GCaMP data. b. Maps based on HbO data.

**Supplementary 3**

***
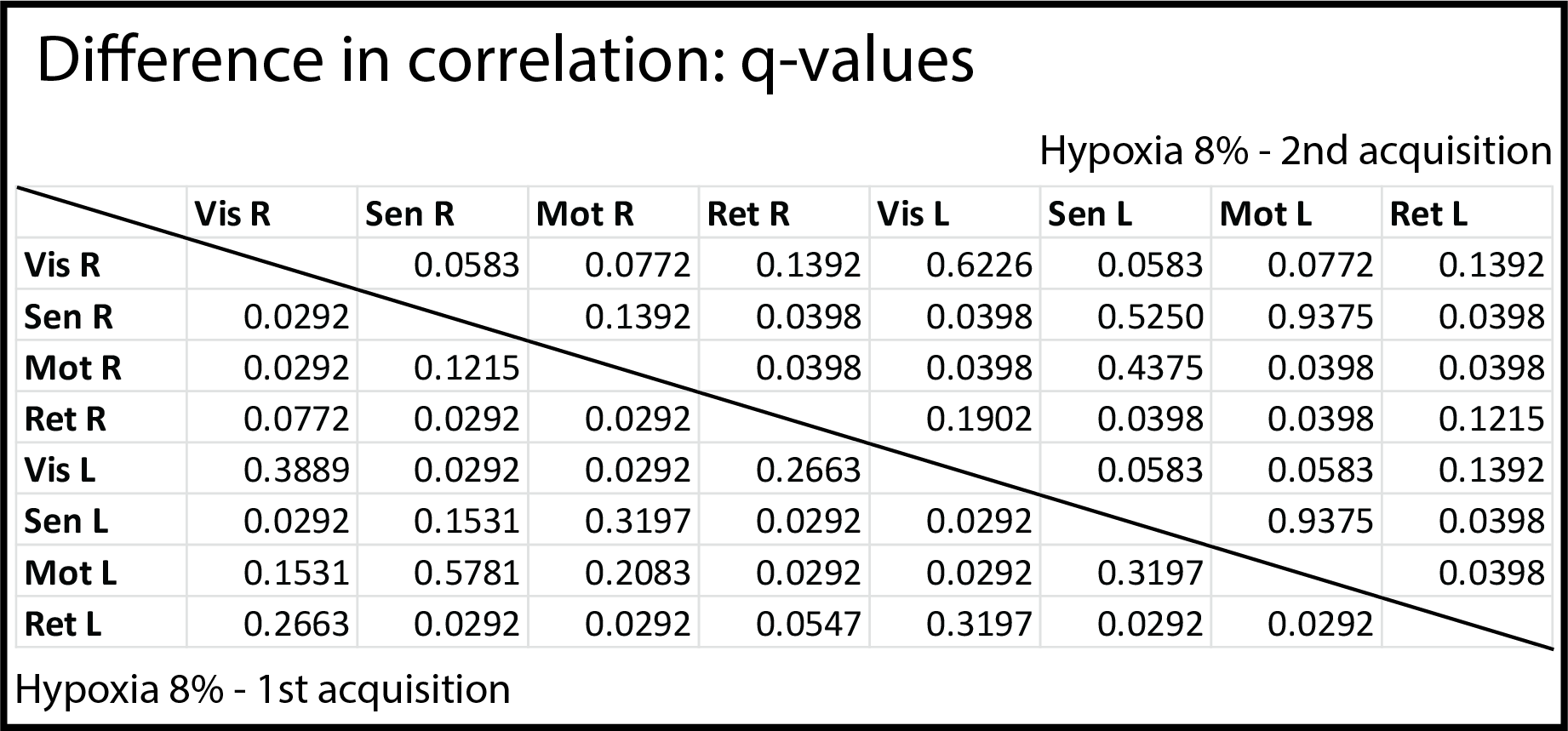
***

Supplementary 3. Q-values for the differences in correlation, based on GCaMP data. Values were computed by comparing correlation z-scores during hypoxia (8% oxygen, minute 12.5 to 17.5) to the period before (minute 2.5 to 7.5). The bottom left triangle shows values for the first acquisition, the top right for the second.
